# The 1,000th Transplant for Multiple Sclerosis and Other Autoimmune Disorders at the HSCT-México Program: A Myriad of Experiences and Knowledge

**DOI:** 10.3389/fneur.2021.647425

**Published:** 2021-02-22

**Authors:** Iván Murrieta-Álvarez, Yahveth Cantero-Fortiz, Andrés A. León-Peña, Juan C. Olivares-Gazca, José Manuel Priesca-Marín, Guillermo J. Ruiz-Delgado, Andrés Gómez-De-León, Elías Eugenio Gonzalez-Lopez, José Carlos Jaime-Pérez, David Gómez-Almaguer, Guillermo J. Ruiz-Argüelles

**Affiliations:** ^1^Centro de Hematología y Medicina Interna de Puebla, Puebla, Mexico; ^2^Facultad de Medicina, Universidad Popular Autónoma del Estado de Puebla, Puebla, Mexico; ^3^Escuela de Medicina, Universidad de las Américas Puebla, Puebla, Mexico; ^4^Benemérita Universidad Autónoma de Puebla, Puebla, Mexico; ^5^Laboratorios Clínicos de Puebla, Puebla, Mexico; ^6^Clínica Gómez Almaguer, Monterrey, Mexico; ^7^Hospital Universitario “Dr. José Eleuterio González”, Monterrey, Mexico

**Keywords:** HSCT, multiple sclerosis, PBSCs, cyclophosphamide, non-myeloablative, outpatient care, autoimmune diseases

## Abstract

After gaining experience conducting both auto and allografts in persons with hematological diseases in the HSCT programs in Puebla and Monterrey, México, this study outlines subsequent program autografting patients with autoimmune conditions. The first transplant in multiple sclerosis was conducted in Puebla on July 5, 2006. From 2015 we increased activity autografting persons with autoimmune conditions in the two campuses of the HSCT-México program: Puebla and Monterrey. By December 6, 2020, patient number 1,000 in the program was autografted. In our experience, a significant reduction in the expanded disability status scale score was achieved in all of the three phenotypes of the disease (from a median of 5.1 to 4.5 points), whereas the response rate (defined as a decrease of at least 0.5 of EDSS score regardless of baseline EDSS, or unchanged EDSS) was 83, 78, and 73% after 12 months in the relapsing-remitting, primary-progressive and secondary-progressive forms of multiple sclerosis, respectively. In addition to analyzing the viability, safety, and efficacy of our method, this study contributes new knowledge to the field of both stem cell transplantation and multiple sclerosis.

## Introduction

As with almost everything in life, the number 1,000 has a special meaning. This study specifically refers to the 1,000th hematopoietic stem cell transplantation (HSCT) procedure conducted for multiple sclerosis (MS), neuromyelitis optica (NMO) ankylosing spondylitis, chronic inflammatory demyelinating polyneuropathy (CIDP), and transverse myelitis, at the HSCT-México program in both campuses, Clínica Ruiz in Puebla and Clínica Gómez Almaguer in Monterrey. Besides the shared experiences with patients, caregivers, physicians, trainees, and specialized staff with the single goal to improve patients' lives, this program resulted in fruitful lessons about conducting autotransplants in specific subpopulations of patients and its conditions, which are summarized below, according to historical order and related achievements.

**Figure 1:**
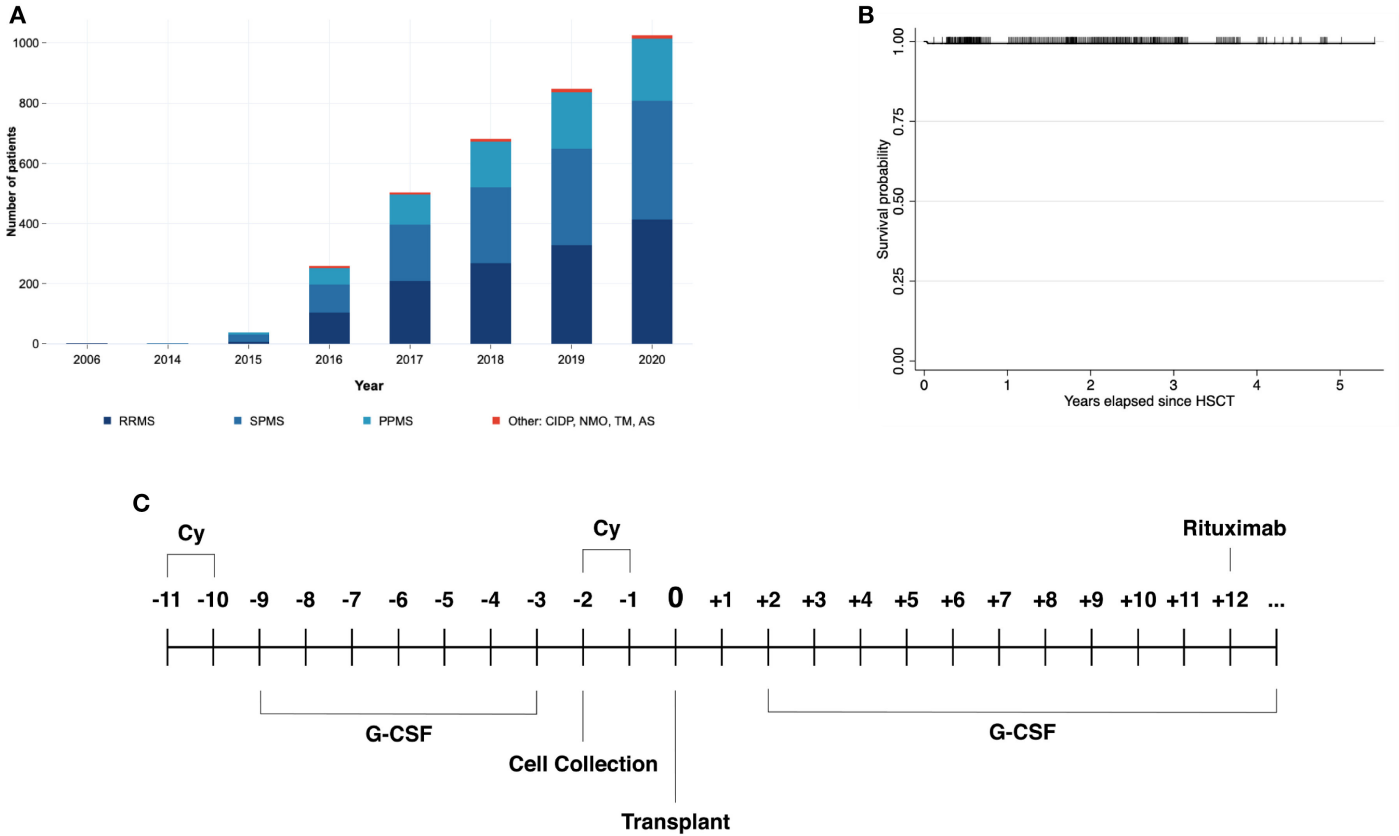
**(A)** Shows the cumulative number of hematopoietic stem cell transplantation (HSCT) procedures in persons with multiple sclerosis, conducted in the two campuses of the HSCT-México program since 2006. **(B)** Depicts a survival function graph according to the Kaplan-Meier method, in patients undergoing HSCT for MS. **(C)** Is a schematic representation of the Mexican conditioning regimen employed for autografting persons with autoimmune disorders. CIDP, chronic inflammatory demyelinating polyneuropathy; TM, transverse myelitis; NMO, neuromyelitis optica; AS, ankylosing spondylitis.

Nowadays the role of autologous HSCT in autoimmune diseases, and especially in MS is well established (1). Several international academic societies have recognized its important role in the treatment of people with MS (2, 3), mainly in the more inflammatory variants of the disease (relapsing-remitting MS). We and others have been researching this area in recent years (4–8). After gaining experience conducting both auto and allografts in persons with hematological diseases in the HSCT programs in Puebla and Monterrey (9), we engaged in autografting patients with autoimmune conditions. The first transplant in multiple sclerosis was conducted in Puebla on July 5, 2006. However, it was until 2015 when we decided to increase autografting autoimmune conditions in the two campuses of the HSCT-México program, Puebla, and Monterrey. By December 6, 2020, we had autografted patient number 1,000 in our program. The main indication of the HSCT's in autoimmune conditions was MS (978 cases), followed by CIDP (16 cases), NMO (3 cases), transverse myelitis (3 cases), and ankylosing spondylitis (1 case), see Figure 1A. The transplants have been reported to the Center for International Blood and Marrow Transplant Research (CIBMTR), and the protocol is registered in ClinicalTrials.gov (NCT02674217).

## Reports on Feasibility and Safety

The pivotal finding of this study is our initial observation regarding the feasibility of the treatment of MS with HSCT using non-frozen peripheral blood stem cells (PBSCs), conducting the procedure on an outpatient basis, and employing a non-myeloablative regimen (10). The main findings were that with this novel method, 286 MS patients given autografts recovered granulocyte and platelet counts within 8 days after the HSCT, very few of them (0.7%) needing red-blood cell transfusions, with overall survival of 100% at 128 mo. These observations indicate that it is possible to perform auto transplants in MS patients employing non-frozen PBSC and outpatient conduction with an outstanding safety profile (10). A subsequent study on a group of 426 patients addressed the safety of the procedure of the HSCT by employing the “Mexican Method,” and confirms the safety of the procedure (11), as previously published (10).

The rationale of splitting the four doses of Cy into two separate blocks is that the initial two doses act both by mobilizing bone marrow stem cells into the peripheral blood and by inducing immunosuppression, whereas the two final doses further damage the autoimmune response, an effect which is consolidated by the high dose-rituximab delivered once the granulocytes have recovered (Figure 1). In our experience in 1,000 MS patients, transplant-related mortality was found to be 2 in 1,000 (0.2%) (see Figure 1B). Other complications were rare as well, for instance, iatrogenic pneumothorax was the most frequent, representing <1% of the total sample, followed by neutropenic fever (0.9%), disease exacerbation (0.5%), and urinary tract infection (0.2%). It is of note that the median length of hospitalization due to complications was 2 days (11).

The salient features of the so-called “Mexican method” of autografting MS patients relies on the use of the total dose of cyclophosphamide (Cy, 200 mg/Kg), delivered two blocks apart, the use of non-frozen peripheral blood stem cells, the conduction of the whole procedure fully on an outpatient basis and the delivery of rituximab, 1,000 mg at the end of the transplant, once the granulocyte count has recovered (Figure 1C) (12). These changes have resulted in diminished toxicity to the bone marrow, the kidney, and the heart of the patients (12). In a longitudinal study evaluating the glomerular filtration rate (GFR) in persons with MS, we found that those undergoing HSCT for MS, had a lower GFR in comparison with healthy controls, indicating that some pre-existing features could limit renal function. After the conditioning regimen, 75% of patients also demonstrated a significant reduction in the GFR in comparison with their baseline level, an effect that continued for 2 weeks. These observations confirm the need to split the doses of Cy to achieve a safe procedure, a modification that also shortens bone marrow recovery (12), decreasing its cardiac toxicity. Concerning this last point, we have found only a single case of Cy-induced acute cardiomyopathy in the group of 1,000 persons autografted for autoimmune conditions, a figure which contrasts with those informed of up to 23% of Cy-related acute cardiac toxicity informed by others (13).

The idea of conducting outpatient HSCT for autoimmune disorders tries to address the cost-effectiveness/safety relation (14, 15). Therefore, to achieve safe and successful conditioning regimens, our group conducted an interventional trial comparing a biosimilar Mexican version of filgrastim with the “standard” chemical patent. This trial confirmed that using the biosimilar version was as efficient as the standard version of filgrastim in terms of leucocyte counts before and after apheresis, granulocyte counts before apheresis, the number of aphereses needed, the number of CD34+ PBSCs obtained per apheresis and grafted to patients, and days to achieve granulocyte recovery (16).

## Reports on Response and Clinical Evaluation

Besides exploring the feasibility and safety of our HSCT method, we have conducted other studies in MS. A contribution from our working party arose from a study evaluating the metabolomic profile of MS patients using an advanced technique to characterize a metabolic pattern measuring alpha-hydroxybutyrate, oleate and insulin, associating them with the level of insulin resistance. The main finding from this study was that the level of insulin resistance was significantly associated with the severity of the disease as measured by the expanded disability status scale (EDSS) score. These observations made an important advance in employing novel technology to define metabolomic signatures in a population with limited interest in this area (17).

Additionally, our group has studied the respiratory function as well in a fairly large sample (466 participants) through forced spirometry. This contribution found that 31% of patients recruited for HSCT, with no important comorbidities and factors related to pulmonary dysfunction had an abnormal spirogram, most frequently with a restrictive pattern. Of note, there was a significant association of predicted values of forced vital capacity and forced expiratory volume with EDSS and disease duration (18).

Having proved that the Mexican HSCT method was both feasible (10) and safe (11), we have recently analyzed data of its efficacy in a group of 617 MS patients (8), this being the largest published single-center experience of HSCT in persons with MS. In this communication, a significant reduction in the EDSS score was achieved in all the three phenotypes of MS (from a median of 5.1 to 4.5 points) 12 months after the transplant, whereas the response rate (defined as a decrease of at least 0.5 of EDSS score regardless of baseline EDSS, or unchanged EDSS) was 83, 78, and 73% after 12 months in the relapsing-remitting, primary-progressive and secondary-progressive forms of MS, respectively. Although this study was limited due to the lack of a comprehensive follow-up assessment, i.e., the EDSS score evaluation at the given time points was achieved in 72% of all patients (240 patients at 1 year, 136 at 2 years, and 19 at 3 years). These results could demonstrate a consistent effect across all groups and period of study (8).

**Figure 2:**
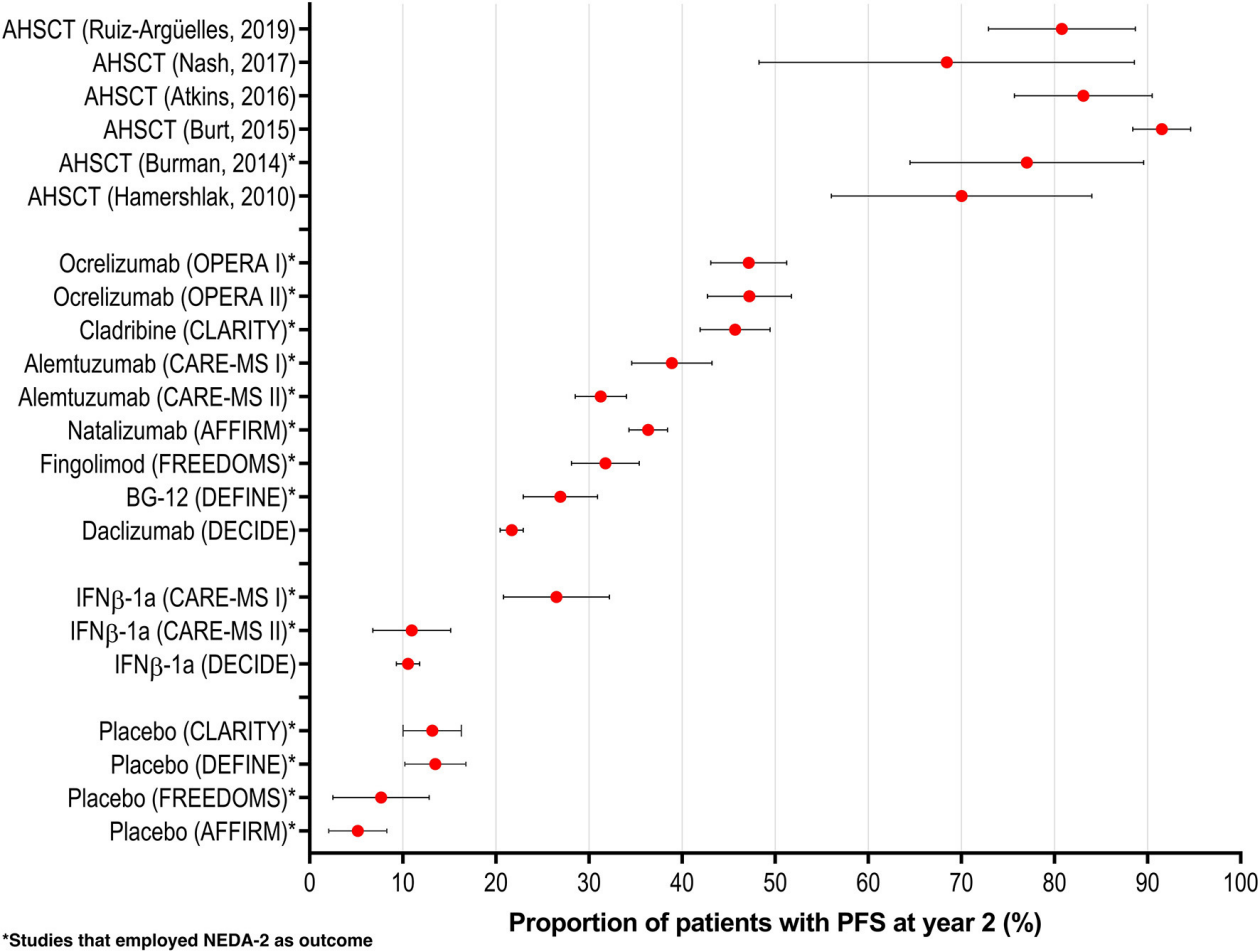
Proportion of patients with a positive response (NEDA-2 or others) at 2 years after autologous hematopoietic stem cell transplantation (HSCT). It is rather clear that all HSCT programs are endowed with better results than those obtained with different drugs. Adapted from (20).

These data also indicate that the failure rate of HSCT in MS employing the “Mexican method” is around 20%, a figure substantially lower than that found using novel, expensive MS drugs (19) and very similar to the figures of other centers grafting MS patients employing other methods (see Figure 2), thus proving the efficacy of the “Mexican method.” Of important note, the long-term follow-up for this cohort is ongoing. Similar to other reports, we have observed fairly similar results and a potential trend of better outcomes in the relapsing form of MS. For instance, a systematic review with meta-analysis (20) of more than 750 patients showed that patients with relapsing-remitting MS had a significantly lower rate of progression and that response at 2 years was 83%. Moreover, Burt et al. (5) showed that HSCT in relapsing-remitting MS induced a significantly higher response in comparison with continued therapy. Interestingly, we have also observed that MS patients who have an early response do neurologically better in the long term than those not showing an immediate response to the HSCT (21). Taking this into account, there are additional potentially beneficial uses of autografting in EM persons; one could be to combine therapeutic schemes including administration of natalizumab or ocrelizumab before and/or after auto-HSCT to benefit from their differentiated effects in restoring the immune system balance on the mostly young productive age patients.

We also prospectively analyzed the mobilization kinetics of CD34+ cells in MS patients employing granulocyte colony-stimulating factor (G-CSF) and cyclophosphamide, an important topic which had not been studied in detail (21), and we found that the CD34+ cell count increased 26-fold after mobilization, and during large volume leukapheresis the number of CD34+ cells in peripheral blood increased from 51.29 CD34+/μL at the start to 62.3 CD34+/μL at the end. These results proved the mobilization with G-CSF and Cy in MS patients as an effective method for CD34+ hematoprogenitor release from bone marrow and intra-apheresis recruitment (22).

Concerning the effects of HSCT on the inflammatory environment of persons with MS we have analyzed the changes in serum cytokines after autologous HSCT employing the “Mexican method.” All patients enrolled had a serum collection 14 days before and 14 after HSCT and IL-6, IL-9, IL-10, IL 17-A, IL-21, IL-22, IL-23, TNF-A, CCL2, CCL3, and CCL4 were measured by magnetic bead-based immunoassay. Interesting results were obtained, detecting that patients had a significant decrease of pro-inflammatory IL-21 (25.9 to 17.0 pg/mL; *p* 0.003) and IL-22 (0.1 to 0.01 ng/mL; *p* 0.028) and a significant increment of inflammatory CCL2 (885.1 to 1170.2 pg/mL; p 0.001) and CCL4 (67.1 to 81.8 pg/mL; *p* 0.039) after HSCT. The impact of this study lies in the fact that the decrease of IL-21 and IL-22 coupled with an increment of CCL2 and CCL4 suggest the immunomodulatory effect of AHSCT and could be another early indicator of its efficacy (22).

## The “Mexican HSCT Method” During the COVID-19 Pandemic

As a result of the changes which we have made to HSCT procedures, not only in MS but also in other hematological conditions (9), we have been able to conduct them even in adverse circumstances such as those stemming from the Coronavirus pandemic (23). Our ability to conduct HSCT on an outpatient basis has also resulted in the feasibility of doing them in unexpected and unique circumstances such as those prevailing nowadays, creating a “COVID-19 free environment” to conduct safely HSCT not only important in MS patients but also in other hematological conditions (23). Control measures designed to prevent COVID-19 outbreaks include travel history screening of all patients, continuous molecular (RT-PCR) testing of all personnel and patients (before and after the grafting), the transient lockdown of patients until confirmation of results, limitation in the number of essential personnel at the center for outpatient patients, strict hand-hygiene protocol, rational use of personal protective equipment (N95-level masks, plastic windows in exam rooms, etc.) for patients, caregivers and healthcare staff. We have shown that keeping patients away from hospitals results in a substantially diminished risk of acquiring COVID-19 and other nosocomial infections. In a group of 126 patients autografted during the COVID-19 era, none acquired the infection during the HSCT conducted both in Puebla and Monterrey, the two campuses of the HSCT-México program.

## Conclusions

The implementation, development, and consolidation of the HSCT-México program for autoimmune diseases in its two campuses (Puebla and Monterrey) has resulted in academic publications and participation in meetings. Between 2015 and 2020, we published 16 papers on our experience in MS in peer-reviewed journals. We have been able to add information about how to conduct HSCT in patients with autoimmune diseases, mainly MS. This procedure is often the best therapeutic option for persons with the disease. Reflecting and building upon the results of other HSCT programs, our experiences have shown promising findings in terms of the relapsing remitting phenotype of the condition, in comparison with several conventional disease modifying therapies (20) (see Figure 2), but the results of our HSCT procedure are also adequate in the other phenotypes of the disease.

## Author Contributions

IM-A, GJR-D, and GJR-A: conceptualization, design, analysis and writing. YC-F, AG-D-L, JJ-P, DG-A, and AL-P: analysis, writing and review. JCO-G, EEG-L, and JP-M: review. All authors contributed to the article and approved the submitted revision.

## Conflict of Interest

The authors declare that the research was conducted in the absence of any commercial or financial relationships that could be construed as a potential conflict of interest.
